# Contributions to the History of Development of the Teeth

**Published:** 1888-07

**Authors:** Carl Heitzmann, C. F. W. Bödecker


### CONTRIBUTIONS TO THE HISTORY OF DEVELOPMENT OF THE TEETH.

BY CARL HEITZMANN, M. D., AND C. F. W. BÖDECKER, D. D. S., M. D. S.

CONCLUDED FROM PAGE 289.

Dr. J. L. Williams (Dental Cosmos, Vol. XXVI, page 193) says, "I have examined many specimens mounted in dilute glycerine, in which the elements of the enamel organ and those of the dentinal germ were in juxtaposition, but without discovering this structureless, transparent membrane. * * * My own observations lead me to believe that these stellate elements are the result of a direct modification of the primitive polygonal elements, and must, therefore, be regarded as strictly epithelial in their nature. * * * Preceding the development of the ameloblasts, or enamel formers, the original prismatic cells break up or divide into round, nucleated corpuscles. * * * From these embryonal corpuscles are developed the enamel forming cells. * * * The material for the formation of the enamel comes through the enamel organ. * * * I announced * * * that the enamel organ was a true secreting organ. * * * So far as appearance teaches us anything, it seems as though the ameloblasts were the active agents in depositing the lime-salts on the periphery of the dentine, and that they gradually recede with the progressive formation of the enamel. * * * My studies of pulp tissue have led to the discovery that the odontoblasts are probably of the nature of multipolar ganglion cells. * * * The odontoblasts, while composed of what is essentially neural matter, are probably at the same time the active elementary agents in secreting the material for the formation and continued integrity of the dentine. * * * The ameloblasts finally become almost obliterated * * * and are probably calcified to form Nasmyth's membrane. * * * "

W. X. Sudduth, (American Syst. of Dentistry, Philadelphia, 1886, page 518) is of the opinion that during the process of amelification there is no conversion of living tissue into enamel, but that the latter, as well as the dentine, is produced by a process of excretion. "The striæ of Retzius are produced by inequalities upon the surface of the enamel prisms." * * * Nasmyth's membrane arises by a metamorphosis of the ameloblastic layer. * * * The breaking up of the enamel organ, as such, over the apex of the forming tooth is a constant accompaniment of the beginning of calcification." The author also describes the nests and buds which arise from the remnants of the external epithelium and the cord of the enamel organ. Sudduth further says concerning the formation of the enamel : "The first change noted is seen over the apex of the papillæ. The protoplasm begins to break up into columns, which stand at right angles to the side of the papilla ; each column contains a nucleus. * * * The development of the dentine always precedes the formation of enamel. The disappearance of the outer tunic occurs about the same time as the beginning of the calcification of the first layer of the enamel, the salts of calcium which are stored up in the meshes of the stellate reticulum only sufficing to furnish material for the very first formed layer of enamel. With the disappearance of the outer tunic and the stellate reticulum as such, the ameloblasts come in direct communication with the rich plexus of capillary vessels, the latter furnishing the lime-salts for the completion of the calcification of the enamel." In regard to the cells of the stratum intermedium, this writer says: "Just what their signification is I am unable to state positively, but from my studies in comparative embryology, I am led to believe that they supply the places made by the increase in the circumference of the enamel, and account for the short prisms seen in ground sections of enamel. * * * The line of ameloblasts that are first formed does not represent the same number of ameloblasts that will finally complete the process of calcification. The outer circumference of the developed enamel is many times larger than that of the first calcified layer. * * * The office of the spheroidal cells in this instance is to develop ameloblasts to supply the places of those which are carried up with the growing tooth. * * * The final calcification in thickness is accomplished after the atrophy of the enamel organ has occurred. It is absolutely essential that the capillary vessels should come in contact with the enamel cells before the process of calcification can be completed. * * * In the development of bone the osteoblasts do not become calcified, but the lime-salts are deposited around the spherical osteoblasts in the form of spherules, increasing in thickness from within outward, and thus approaching one another, they coalesce. The osteoblasts persist as the organic contents of the lacunæ. * * * In the calcification of dentine the odontoblasts do not become directly calcified, but send out rod-shaped fibrils, around which tubular dentine is formed; so also in the enamel we have the prismatic ameloblasts superintending the deposit of prismatic enamel. * * * Tomes processes, I consider as mechanically made."

Otto Walkhoff (Deutsche Monatsschr. f. Zahnheilkunde, 1887, pages 246 and 304), after giving a good description of the literature on our subject, describes the development as follows: "The basis-substance of the dentine is produced by a transformation of the odontoblasts, as stated by Waldeyer, which is accomplished in such a manner that the cell contents of the periphery of the odontoblast is changed into a homogeneous substance, which is pierced by the dentinal fibers and their transverse offshoots, which latter are formed by the lengthening of the nucleus of the odontoblast." * * * From my observations I have come to the conclusion that the basis-substance is formed in an unbroken contiguity by the pulp cells after forming odontoblasts, without leaving a trace between the single cells, as long as the formation of the dentine is continuous. In later periods of life, however, when the formation of dentine is slow, we observe the contours of the transformed odontoblasts.

L. A. Weil (Deutsche Monatsschr. f. Zahnheilkunde, 1887, page 81, and 1888, page 10), in describing the anatomy of the pulp, states that the zone of dentine which has been calcified last is made up of globular masses. He also observed the structureless zone which is present between the odontoblasts and the newly formed dentine. He further says that the globular arrangement of the dentine can only be observed in newly formed tissue, while the dentine of adult teeth is regular. This author also states that from a morphological point of view the odontoblasts are identical with the ameloblasts, from which the former cannot be distinguished.

---

The writers regret to state that they have been unable to obtain all the publications on this subject. Among the works missing are the important ones of Hannover, Czermack, etc.

